# Summation of perceptual cues in natural visual scenes

**DOI:** 10.1098/rspb.2008.0692

**Published:** 2008-07-15

**Authors:** M To, P.G Lovell, T Troscianko, D.J Tolhurst

**Affiliations:** 1 Department of Physiology, Development and Neuroscience, University of CambridgeDowning Street, Cambridge CB2 3EG, UK; 2 Department of Experimental Psychology, University of Bristol12a Priory Road, Bristol BS8 1TU, UK

**Keywords:** natural scenes, vision, perception, Minkowski summation, cue summation

## Abstract

Natural visual scenes are rich in information, and any neural system analysing them must piece together the many messages from large arrays of diverse feature detectors. It is known how threshold detection of compound visual stimuli (sinusoidal gratings) is determined by their components' thresholds. We investigate whether similar combination rules apply to the perception of the complex and suprathreshold visual elements in naturalistic visual images. Observers gave magnitude estimations (ratings) of the perceived differences between pairs of images made from photographs of natural scenes. Images in some pairs differed along one stimulus dimension such as object colour, location, size or blur. But, for other image pairs, there were composite differences along two dimensions (e.g. both colour and object-location might change). We examined whether the ratings for such composite pairs could be predicted from the two ratings for the respective pairs in which only one stimulus dimension had changed. We found a pooling relationship similar to that proposed for simple stimuli: Minkowski summation with exponent 2.84 yielded the best predictive power (*r*=0.96), an exponent similar to that generally reported for compound grating detection. This suggests that theories based on detecting simple stimuli can encompass visual processing of complex, suprathreshold stimuli.

## 1. Introduction

Our visual world encompasses a rich combination of cues: size, shape, colour, lightness, motion, depth and others. Feature integration binds all this information together, so that we have a useful representation of the natural visual environment (von der Malsburg 1995; Treisman 1996; Ghose & Maunsell 1999; Wolfe & Cave 1999). Straightforward combination rules for neural channels have been proposed for object detection (e.g. Ennis *et al*. 1988) and salience (e.g. Koch & Ullman 1985; Shepard 1987; Li 2002), but these have generally been demonstrated only for simple visual images where the information content is fully specified (e.g. Shepard 1964; Graham 1989; Koene & Zhaoping 2007; Zhaoping & May 2007). Here, we investigate whether such simple combination rules also apply to complex, naturalistic images containing recognizable objects and scenes.

The first factor in discrimination tasks is detectability. Campbell & Robson (1968) first proposed a multiple-channel model whereby visual input at any location is processed by several parallel neural channels, each responsive, e.g. to different stimulus orientations and spatial frequencies. It has since been demonstrated many times that, the more channels a composite visual stimulus activates, the greater the probability that the stimulus will be detected and the lower is its detection threshold (e.g. King-Smith & Kulikowski 1975; Tolhurst 1975; Graham 1977; Robson & Graham 1981). In experiments where the stimulus consists of two or more component sinusoidal gratings, the detectability of the compound stimulus can be estimated by a nonlinear (weighted) summation of the detectability of its components. The summation rule derives from Quick's (1974) pooling function, otherwise known as Minkowski summation (Shepard 1964); it is given by

(1.1) $$S_{\text{c}}={\left(\sum_{i=1}^{n}S_{i}^{m}\right)}^{1/m},$$

where *S*_c_ is the sensitivity (reciprocal of threshold contrast) for the compound stimulus, *S*_*i*_ is the sensitivity to each component stimulus, *n* is the number of components and *m* is the summating Minkowski exponent. Robson & Graham (1981) showed that a Minkowski exponent of approximately 3.5 yielded the strongest predictions in a number of grating detection tasks. Similar values have been reported in many other summation experiments and models based on simple visual stimuli such as lines and Gabor grating patches (e.g. Watson & Nachmias 1980; Watson 1982; Wilson & Gelb 1984; Bonneh & Sagi 1998, 1999; Meese & Williams 2000; Meinhardt & Persike 2003; Watson & Ahumada 2005). The useful applicability of Minkowski summation to predicting thresholds for composite stimuli is clear, but the mechanism is only hypothesized. Detection is probabilistic; the summation rule has generally been interpreted as describing *Probability Summation* (Quick 1974; Graham 1989), although this has been debated (see §4). The probability of detecting a composite stimulus (*P*_c_) would be calculated from the probabilities of detecting the *n* components (*P*_*i*_) independently,

(2.1) $$P_{\text{c}}=1-\prod_{i=1}^{n}\left(1-P_{i}\right).$$

The Minkowski parameter (*m*) is then interpreted as a measure of the slope of the psychometric probability function (Robson & Graham 1981) that relates probability of detection to the contrast (or intensity) of the stimulus.

Rohaly *et al*. (1997) have extended the applicability of Minkowski summation to the detection of objects against backgrounds in images of natural scenes. They found that summating the absolute differences between the background alone and the background with target using a Minkowski exponent of 4 could generate good predictions about the detectability of the target. We have also had some success in modelling the detectability of morphed changes in the shape, texture and colour of naturalistic images of objects and faces (Párraga *et al*. 2005; Tolhurst *et al*. 2005; Lovell *et al*. 2006). While earlier detection experiments combined cues in the same feature dimension (e.g. spatial frequency), these two experiments demonstrate the applicability of the Minkowski combination rule across feature dimensions. These observations therefore illustrate the potential relevance of the Minkowski summation model to more complex visual stimuli. Yet the question remains whether this straightforward but powerful model of detection processes can be extended to even more realistic viewing situations, where the natural images contain differences that lie comfortably *above* detection and discrimination thresholds.

Then, perhaps, we would be investigating not detectability but saliency, another factor that must be considered when discussing perception of natural scenes that contain noticeable changes. Saliency refers to how much an object contrasts from its surrounding, thereby attracting attention to itself (Titchener 1908). Koch & Ullman (1985) proposed that features are first processed independently and then summed up at a later stage to form a salience map. However, Li (2002) designed a V1 model based on the physiological and anatomical properties of V1 neurons and suggested that saliency is determined at an earlier stage by the most active V1 cells, i.e. the location of the visual field eliciting the strongest response from V1 neurons will most probably be selected for further attentional processing. Recent visual studies have supported the V1 model: reaction times for locating a target (or texture border) are better explained using this maximum rule than by a simple summation rule (Koene & Zhaoping 2007; Zhaoping & May 2007). Furthermore, in the context of natural images, Lewis & Zhaoping (2005) report that a maximum rule is more accurate in predicting salient locations in a database of natural scenes. Because a maximum rule is equivalent to Minkowski summation with power of infinity, this reinforces the potential significance of the Minkowski summation model in our investigation.

In this paper, we ask human observers to rate the perceived *supra*threshold differences between pairs of images made from the photographs of natural scenes. In particular, we examine the perception of the difference between paired scenes that contain two visible and recognizable differences (e.g. differences in blur or colour or object size or location), and ask how the perception of these composite differences relates to the perception of image pairs where there is only one difference. Our experiments differ from those described above in several aspects. First, by contrast to most studies, our stimulus sets are composed of hundreds of complex naturalistic images, a step towards studying vision in the real world. Second, the image differences presented in these experiments are not only substantially above threshold, but also span across a wide range of categories, e.g. colour, blur, shape change, etc. This allows us to investigate how a larger array of cues integrate in a more realistic set up. Third, unlike the detection and saliency experiments, no thresholds or reaction times are recorded: our observers are asked to enter ratings that indicate how they perceive differences between the images. Our ratings experiments will show that a Minkowski summation rule describes the relations very well.

## 2. Material and methods

### (a) Display apparatus

Stimuli were presented on a 19″ SONY CRT display driven with 800×600 pixels and a frame rate of 120 Hz by a ViSaGe system (Cambridge Research Systems). The display was viewed in a darkened room from 2.28 m, so that the visible area subtended 10 by 7.5°; each square pixel subtended 0.75 min. The stimuli were square (256×256 pixel) coloured images constructed from digitized photographs of natural scenes, occupying 3.2° square in the centre of the display. Each pixel in the stimuli was represented with eight bits each of red, green and blue, and the pixel values were fed through linearizing look-up tables to be displayed through 14 bit DACs; thus, each colour plane was presented with 256 equally spaced precise luminance steps (Pelli & Zhang 1991). When and where the display was not occupied by a stimulus, it was held at a mid-brightness grey (55 cd m^−2^; CIE *x*,*y* 0.28, 0.29), except for a small dark fixation dot in the centre of the screen. The fixation dot was extinguished when stimuli were present. The brightest white pixel across the stimuli had a luminance of 110 cd m^−2^.

### (b) Construction of stimuli

Images of natural scenes were captured using three digital cameras, as follows: a Nikon Coolpix 950 (1600×1200 pixels), a Nikon Coolpix 5700 (2560×1920 pixels) and a JVC GR-DVL-9700 digital camcorder. The details are described in the electronic supplementary material.

In experiment 1, images were separated into six broad and partly overlapping thematic categories, as follows: animals, landscape, objects, people, plants and garden or still-life scenes. Each category contained 30 parent images each matched with 5 variants, to make up 900 different image pairs in total. Examples of parent images and image pairs can be seen in figures 1*a* and 3. For 325 of the pairs, the variant was a second photograph of the same scene taken when, say, an object had moved or when changes in the illumination had changed the shadowing. Other variants were made from originals using PaintShopPro (JASC software) or code written in Matlab (The Mathworks). In some variants, part or the entire scene could be blurred to varying degrees, or the hue and saturation of objects or the whole scene could be changed, while leaving the brightness relatively unaffected. Objects could be ‘painted out’ or, by cut-and-paste they could be duplicated or moved within scenes.

The 900 image variants were designed to test a number of different models or hypotheses (e.g. Lovell *et al*. 2005, 2006). However, for the present purpose, many of the variants contributed to 136 *combination sets*. Each combination set was made up of three image pairs based on a single parent image (see figure 1*a*): in two pairs, the images differed from the parent along different single dimensions (e.g. object colour, location or presence, size, blur); the third image pair was a composite, differing along both the dimensions. Some image pairs contributed to more than one combination set.

In experiment 1, observers were presented with natural scene images. For experiment 2, we wanted to present stimuli with the same spatial and colour complexity as the natural ones, but with the semantic content difficult to discern. Inverting or negating images of faces or objects makes them difficult to recognize (e.g. Yin 1969; Bruce & Langton 1994; Haxby *et al*. 1999; Rossion *et al*. 2002; Vuong *et al*. 2005). We were able to present images upside down, but making negatives (subtracting each pixel value in each colour plane from 256) was not successful, because it made the images look desaturated. Instead we made pseudo-negatives as follows. The R, G and B planes were processed separately. In each plane the pixel values were ranked and then the ranking was reversed, before the three reversed planes were combined again to form the ‘modified’ image that resembled the negative of the original. This image was then inverted. Some examples are shown in figure 1*b*. There were, of course, 136 combination sets in this experiment as well.

The stimulus set for experiment 3 contained 588 image pairs, including 432 combination sets (many of the image pairs contributed to several combination sets). These were made from only six parent images; for each parent there were 48 variants. Starting with a parent image, variants could be made by changing, say, the size or colour of an object in six steps. Also, variants could be made by changing the contrast of shadows in six steps. It was possible to construct 49 images from the parent, including those where there was change in one (or neither) of the two dimensions. For any given change in one dimension, it was possible to make an image variant that had seven different values (including no change from the original) in the second dimension. Figure 1*d* shows some examples from one of the six families. In this experiment, each of the 294 image pairs was presented once upright and once inverted, the order of presentation of upright and inverted images being randomized.

### (c) Procedure

#### (i) Experiments 1 and 2

Difference ratings were collected for 900 upright natural scene image pairs from each observer (see the electronic supplementary material), who was initially instructed during a quick demonstration session, where he/she was shown the different types of differences that could be presented to him/her. A training session then followed the demonstration programme. In this phase, observers were asked to rate 51 image pairs presented in a random order. All images used in the demonstration and training phases were different from those to be used in the testing phase proper. During the demonstration and testing phases for experiments 1 and 2, observers were repeatedly presented with the same standard ‘lily’ image pair (figure 1*c*), whose magnitude difference was defined as ‘20’. They were instructed that their ratings of the subjective difference between any other image pair should be based on this standard pair: if they perceived the difference between the test pairs to be lesser, equal or greater than the standard pair, their ratings should be less, equal or greater than 20, respectively. They were instructed to use a ratio scale so that, if a given image pair seemed to have a difference twice as large as that of the reference pair, they would assign a value twice as large to that image pair (in this case, 40). No upper limit was set so that observers could rate the differences as highly as they saw fit. Observers were also told that sometimes image pairs may be identical and, in such cases, they should set the rating to zero.

The testing phase was divided into 6 blocks of 150 image pairs. The image presentation sequence was random so that a given combination stimulus might have been presented before or after one or both of the single-change stimuli; the order was randomized differently for each observer. Each block started with the presentation of the standard lily image pair, and this standard was regularly presented after every 15 trials to remind the observers of the standard difference of 20. On each trial, the fixation dot was extinguished and a randomly selected image from the current image pair was presented for 833 ms; then the fixation point was presented in the centre of the grey screen for 83 ms; then the other image from the image pair was presented for 833 ms; the fixation point was presented again for 83 ms, and finally the first image shown within the current trial was presented again (833 ms). Observers were asked to gaze at the central fixation point between image presentations and to maintain this fixation during image presentations. The 83 ms interval was long enough that observers could not gain any cue about potential image differences from apparent motion of objects in changed positions. The featureless display during the interval was not intended to be a distractor and the image changes were generally clear and unsurprising. Although the presence of the blank interval made the changes in some image pairs harder to detect, we were not trying to imitate some kinds of ‘change blindness’ paradigm (Simons & Rensink 2005) where large changes are disguised by the nature of the image transition or interval; once recognized, those changes become easy to see. However, despite the interstimulus interval, changes presented in the following experiments were generally noticeable and easily identifiable.

Following these presentations, a random number between 10 and 30 appeared at the centre of the screen, and the observers were asked to modify this number using a Cambridge Research Systems CB6 response box until their choice of difference rating was reached.

#### (ii) Experiment 3

The procedure was essentially identical, except that a different standard pair was used, one more thematically similar to the landscape or garden scenes in the experiment. The experiment was conducted in 4 blocks of 147 trials.

### (d) Data collation

In each of the three experiments, the ratings of the observers were averaged together for further analysis. Typically, the median rating given by each observer over the whole experiment was approximately 20. The results for each observer were first divided by their median value and the ratings were rescaled to give a median of exactly 20. Then, the scaled ratings of the several observers were averaged together, typically with standard errors of approximately 2.5.

## 3. Results

Observers were presented with pairs of coloured images derived from photographs of natural scenes, and were asked to give numerical magnitude estimates (Stevens 1975; Gescheider 1997) of how different the images in each pair seemed to be. The robustness of these measures of visual performance is assessed in the electronic supplementary material. The aim of the experiments was to determine how the visual system combines multiple cues in order to provide a single judgement about natural image difference. The experiments were based around *combination sets*. Starting from a single reference image, the observers rated the perceived difference between that image and three others (e.g. figure 1*a*). In the first pair (a component pair), the images might differ in one dimension such as colour; in the second pair (a second component), the images would differ in a second dimension such as object shape; in the final pair (the composite), the images would differ in both the dimensions. All image pairs were presented in a different random order for each observer. We averaged the ratings given by 11 or more observers for each image pair, and we examine below how the ratings to two component changes are combined to give a rating for the composite stimulus.

Results are evaluated from three different experiments. In experiment 1, 11 observers were presented with a wide variety (900) of image pairs, which mostly looked like normal digitized photographs (e.g. figures 1*a* and 3) that included 136 combination sets. In experiment 2, we repeated the procedure using the same image-pairs but after they were both inverted and colour distorted (figure 1*b*) on 11 new observers; the inversion and colour distortion were intended to allow us to examine the role of semantic context and higher-level features. In experiment 3, we examined summation of stimulus dimensions in a more systematic way, testing 15 observers on 432 further combination sets (both normal and inverted), generated from six parent images by summing coupled cues in various proportions (figure 1*d*).

Figure 2 examines how well several different combination rules were able to predict the measured rating (*R*3) to the composite stimulus in each combination set from the separate ratings (*R*1 and *R*2) to its two component image pairs. Figure 2*a*(i)–*c*(i) shows the simplest prediction: that the rating to the composite image is the simple arithmetic sum of the ratings to the two component images. Clearly, for each of the three experiments (figure 2 rows *a–c*), arithmetic addition of the two component ratings predicts a composite rating that is substantially higher than that actually measured. We also examined whether the composite rating could be predicted as the mean of the two component ratings (graphs not shown), but the fit was also poor. For all rules, the Pearson correlation coefficient was above 0.9 (table 1), but this shows only that experiment and prediction were proportional and not that they were identical. Table 1 lists the sum of squared deviations between measured and predicted *R*3 for all experiments and putative summation rules, as a direct measure of goodness of fit between experiment and prediction. Figure 2*a*(ii)–*c*(ii) shows how well the measured rating to the composite is matched by the maximum of the two individual ratings to the component stimuli. The match (see table 1) is very much better than for the arithmetic sum, but the maximum slightly underestimates the measured value of *R*3 in all experiments.

The rightmost column of figure 2 shows that Minkowski summation of the two component ratings gives a good prediction of the actual rating to the composite stimulus. Minkowski summation (equation (1.1)) is widely used to model how the detection thresholds of simple and complex visual stimuli depend on the thresholds for the stimulus components (e.g. Stromeyer & Klein 1975; Mostafavi & Sakrison 1976; Quick *et al*. 1978; Robson & Graham 1981; Rohaly *et al*. 1997; Watson & Solomon 1997; Párraga *et al*. 2005; Watson & Ahumada 2005; Lovell *et al*. 2006) and has been proposed as the basis of a ‘general law’ of sensory encoding (Shepard 1987). We have examined whether an analogous summation rule applies to the perceived differences between naturalistic images,

(3.1) $$\text{predicted}\;R3={\left(R1^{m}+R2^{m}\right)}^{1/m},$$

where *m* is the Minkowski exponent. It will be noted that an exponent of unity is simple arithmetic summation (or ‘city-block summation’), an exponent of 2 is the Euclidian distance, while the maximum is given by a high exponent (Lewis & Zhaoping 2005; Koene & Zhaoping 2007; Zhaoping & May 2007). For each of the three experiments separately, we used an iterative search to find the value of the exponent which minimized the sum of squared deviations between the predicted value of *R*3 and the measured value. Figure 2 and table 1 show that, for the best-fitting exponents, Minkowski summation gives a good prediction of the composite ratings. Figure 3 shows pictorially how the Minkowski summation rule predicts the rating to the composite stimuli for four combination sets from experiment 1.

Figure 4*a* plots the measured and predicted ratings for all 704 combination sets, pooled across the three experiments. The single best-fitting value of the Minkowski exponent is 2.84, very similar to values that best describe detection experiments with simple visual stimuli (Robson & Graham 1981; Watson & Solomon 1997; Watson & Ahumada 2005; and figure 4*d*, see §4).

However, when the ratings to the component stimuli (*R*1 and *R*2) are very different, it will be noted that the predictions of the arithmetic summation, maximum and Minkowski summation rules will all be nearly the same, i.e. the predicted value of *R*3 will be approximately the same as the bigger of the two values *R*1 and *R*2 in all cases. Indeed, the fits of the maximum rule and the Minkowski summation rule are not very different in the combined dataset (table 1). The graphs in figure 2 are therefore not as stringent a test of the summation rules as we would like. To verify that the Minkowski combination is indeed the most effective model for feature pooling, we discarded all those combination sets in which *R*1 and *R*2 differed by more than a factor of 1.4, leaving 208 sets where the two component ratings were of similar magnitude. Figure 4*b* plots the measured and predicted ratings for the composite stimuli of these remaining sets, calculated with a best-fitting Minkowski exponent of 2.98. Table 1 shows that, after discarding the combination sets that have little predictive power, the sum of squared deviations per point has decreased slightly for the Minkowski summation rule, but it has *increased* more substantially for the maximum rule. This does suggest that the Minkowski summation rule is a better description of the pooling strategy than the maximum rule.

Figure 4*c* shows how the sum of squared deviations between measured and predicted *R*3 depends upon the exponent for the selected dataset of 208 combination sets; similarly shaped graphs were found for the 3 individual experiments and for the overall dataset of 704 combination sets. There is an asymmetric minimum, which is shallow on the higher side, where detection models often place the exponent (Robson & Graham 1981; Rohaly *et al*. 1997; Watson & Solomon 1997; Párraga *et al*. 2005; Lovell *et al*. 2006).

## 4. Discussion

The visual world is a rich amalgam of information, and the role of the visual system is to integrate all the pieces of information together to build coherent percepts from the component pieces. How are the various spatial and chromatic cues in natural scenes pooled to give unified percepts? Previous research, with simple geometric figures (Shepard 1964, 1987) or sinusoidal gratings (see Graham 1989), has demonstrated the applicability of several different combination rules, one of which is Minkowski-weighted summation. The similarity between one composite stimulus and another, or the detection sensitivity for a composite grating target is given by raising the contribution of each component to some power, and then summing the result (see equations (1.1) and (3.1)). Shepard (1964, 1987) proposed that, in a wide variety of sensory tasks, the summation power would be either 2 (Euclidian summation) or 1 (‘city-block’ summation). In fact, many detailed studies of the detectability of compound sinusoidal gratings have found the summation power to be higher than this, generally in the range 3–4 (Watson & Nachmias 1980; Robson & Graham 1981; Watson 1982; Wilson & Gelb 1984; Watson & Solomon 1997; Bonneh & Sagi 1998, 1999; Meese & Williams 2000; Meinhardt & Persike 2003; Watson & Ahumada 2005). Furthermore, the same summation rule with the same value of exponent has been used in complex models of the detection of targets in natural visual scenes (Rohaly *et al*. 1997) and the detection of spatial or spatiochromatic changes in morphed images of natural objects (Párraga *et al*. 2005; Tolhurst *et al*. 2005; Lovell *et al*. 2006). It is therefore perhaps no coincidence that a power rule of 3–4 should be found in this experiment where suprathreshold cues are integrated.

Figure 4*d* shows the results of some of our own experiments on the detectability of composite sinusoidal grating stimuli (see figure legend for details). The observers detected Gabor patches of grating presented either singly or in pairs, one component to each side of the central fixation spot (cf Meese & Williams 2000). The graph shows the measured sensitivity to the composite stimulus (both Gabors presented together) on the *x*-axis, plotted against the sensitivity predicted from the sensitivities to the two component Gabors presented singly. The line shows the good relation that holds for Minkowski summation of the component sensitivities, with exponent of 1.97, on the lower range of values previously reported for grating summation.

Now, natural scenes comprise many elements that would seem to be more complex than just the sum of a few simple features, and many of those components are considerably above detection threshold (Chirimuuta *et al*. 2003; Clatworthy *et al*. 2003). Processing of natural scenes presumably involves activation of numerous channels from which pooled information needs to be extracted. It is therefore important to establish whether the simple rules that govern the combination of channels when the stimuli are simple will generalize to these more complex images, higher contrasts and more natural discrimination tasks. Our image-difference rating task is a suitable candidate for study because it is a realistic task for an observer, but is complex enough to permit the elucidation of visual combination rules.

Thus, the purpose of the present experiments was twofold, as follows: (i) to examine whether the Minkowski summation model can be extended to these more realistic conditions and tasks, where complex natural images contain salient differences and people have to judge their magnitude; (ii) to investigate whether the mechanisms that underlie suprathreshold summation are similar to those in detection tasks with simple stimuli. We examined a number of combination rules that might determine the magnitude rating that an observer gives to a natural scene stimulus in which there are two feature changes. Simple summation of the ratings to the separate feature changes (city-block summation) failed badly. Euclidian summation (not illustrated) would have fared better, but our experimental results show that the most predictive combination rule in several experiments was Minkowski summation, specifically with exponents, as follows: 2.78 (experiment 1), 2.79 (experiment 2), 2.95 (experiment 3) and 2.84 (all experiments combined). These values are very similar to those reported in previous threshold-level experiments (3–4, see above). The present analysis attempts to predict the magnitude ratings that observers give to composite image differences, given that we already know the ratings that they have given to the two components separately. We have also tried to construct biologically realistic models of visual cortex processing that would allow us to explain the ratings that observers give to any arbitrary image pair from first visual principles: from knowing how populations of V1 simple cells with different orientation and spatial-frequency tuning might respond to the stimuli, and these models too work best when the contributions of the individual simple cells are summed with Minkowski exponents close to 3 (Lovell *et al*. 2006; To *et al*. 2007). These findings could imply a generalized feature integration mechanism that may be underlying a whole variety of stimuli, from supra threshold elements in naturalistic images to detection in threshold grating experiments.

Early studies of grating detection thresholds supposed that the Minkowski summation rule reflected a particular mechanism, i.e. probability summation (Graham 1977; Graham *et al*. 1978; Quick *et al*. 1978; Robson & Graham 1981). If the detectability of each component was independently probabilistic, then the composite stimulus would be detected more frequently than any component because, in effect, the extra components increase the chance that at least one of them will be detected (see equation (2.1)). The Minkowski exponent of 3–4 in those early experiments was then interpreted as a measure of the slope of the psychometric function (often fitted as a Weibull function whose parameter had the same value as the Minkowski exponent), which relates probability of detection to the logarithm of contrast (Quick 1974). Although the arithmetic of probability summation has worked well in many circumstances, it has not often been formally demonstrated that the component elements within the stimulus are indeed detected probabilistically and independently (but see Tolhurst 1975 in the time domain). Some have argued that the incomplete summation results from lateral interactions between similarly tuned channels (Bonneh & Sagi 1998, 1999; Meinhardt & Persike 2003; Meinhardt *et al*. 2004, 2006). We do not think that cue summation in our present suprathreshold rating task is easily interpreted in probabilistic terms, suggesting that the Minkowski summation rule, while covering both threshold and suprathreshold levels, is not necessarily fixed to the probability summation idea.

We should point out that while the best predictions were obtained with a Minkowski exponent of approximately 3, we did find that the maximum rule also yielded very good predictions. These results are consistent with Li's (2002) V1 model and the saliency experiments described earlier (Koene & Zhaoping 2007; Zhaoping & May 2007). The maximum rule has no free parameters (the implicit Minkowski exponent is fixed at infinity), and perhaps the superior performance of our Minkowski rule is partly because the fitting parameter is free to change. However, removing one degree of freedom from the analyses with hundreds of data must make little difference to its advantage.

Over 20 years ago, Shepard (1987) suggested a universal law for psychological processing, which he hoped would be as applicable as the laws of Newton or Einstein. According to Shepard, this law of generalization for psychological science involved Minkowski summation of cues from individual components in a stimulus and would hold true ‘across perceptual dimensions, modalities, individuals, and species.’ His paper discussed, among other things, how simple visual stimuli were processed according to size, lightness, saturation, spectral hues, shapes and position, and combinations of these. Our experiments might be seen as a justification of this ‘law’ in the context of viewing natural visual scenes. Furthermore, Shepard proposed that the ‘universal law’ would be applicable to other sensory modalities such as audition. We have investigated whether similar cue summation can indeed be obtained in audition by extending our present paradigm: subjects were asked to rate the difference between two musical sequences (approx. 2 s long) instead of two visual images. These sequences could differ along one or two of the following dimensions: loudness, scale, the appearance or disappearance of single notes and instrumental timbre. Remarkably, preliminary results have demonstrated that integration of auditory cues follows a combination rule similar to the visual case: Minkowski summation with exponent 2.95 generated the best predictions for combined changes (*r*=0.864; *n*=96; To *et al*. in preparation).

Given the possibility of a universal ‘Minkowski summation Law’, we should ask what it is about the sensory stimuli in the natural world or the coding mechanisms in the nervous system that makes such a rule appropriate and, particularly, why exponents in the range 2–4 are so often found. One answer might lie in the degree to which the responses of different sensory neurons are correlated when stimulated by natural scenes (Field 1987; Schwartz & Simoncelli 2001; Lewis & Zhaoping 2005); one strategy in the design of sensory systems might be the reduction of coding redundancy, i.e. reduction in correlations between neuronal responses (Srinivasan *et al*. 1982; Atick & Redlich 1992). If the responses provided by two neurons about cues are utterly uncorrelated, it might be appropriate to sum those cues because each neuron conveys a uniquely important signal (Minkowski exponent of one); but if the neurons' responses are highly correlated, we need to consider the information given by only one of them (the maximum rule, or Minkowski summation with exponent of infinity). Given that recordings and computational models of paired visual neurons show that their responses to natural scenes have some small correlation one with another (e.g. Vinje & Gallant 2000; Schwartz & Simoncelli 2001; Schneidman *et al*. 2006), it may indeed be appropriate that the ‘universal’ value of the Minkowski summation exponent is a little greater than unity but a lot lower than infinity.

## Figures and Tables

**Figure 1:**
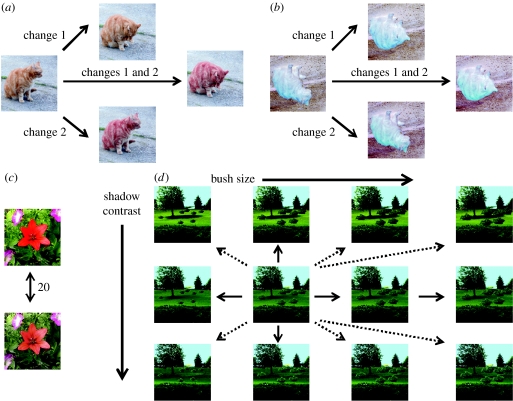
(*a*) some examples of image pairs used in experiment 1. Three pairs are shown, constituting one combination set: starting from a single reference image, the comparison image could vary in either of the two stimulus dimensions or in both. (*b*) The same pairs used in experiment 2, after they had been inverted and the pixel colours distorted. (*c*) The standard pair used in experiments 1 and 2; the difference between these two images was defined as having a magnitude of 20. (*d*) Sample combination sets from one of six families used in experiment 3. Starting from a single reference image, the contrast of the shadows could be changed to six new levels (two are shown here), and the size of the bushes could also be changed to six new levels (three are shown here). Altogether there could be 49 images, including the reference.

**Figure 2:**
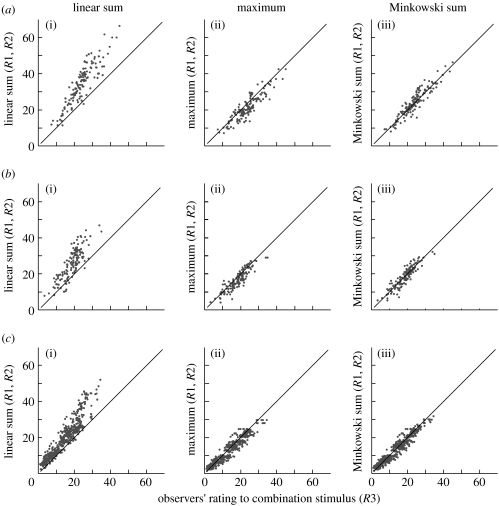
Predictions of the rating (*R*3) given to the composite image pair in a combination set from the individual ratings (*R*1 and *R*2) to the two separate component images. The results from experiments 1, 2 and 3 are presented in (*a*(i–iii)),(*b*(i–iii)), (*c*(i–iii)), respectively. (*a*(i)–*c*(i)) The arithmetic sum of *R*1 and *R*2 plotted against the measured *R*3; (*a*(ii)–*c*(ii)) the maximum of *R*1 and *R*2 plotted against *R*3; (*a*(iii)–*c*(iii)) the Minkowski sum (equation (3.1)) of *R*1 and *R*2 plotted against *R*3 (Minkowski exponents: 2.78, 2.79 and 2.95 in (*a*–*c*, respectively). Lines of equality are shown; details of the fits are given in table 1.

**Figure 3:**
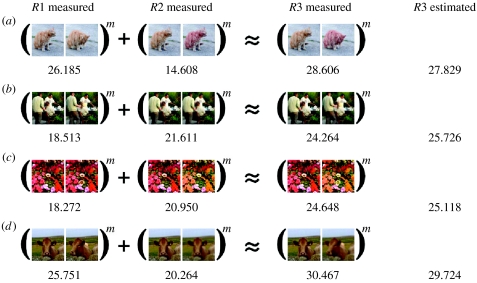
(*a*–*d*) In experiment 1, ratings of pairs with single changes (*R*1 and *R*2) were combined using a Minkowski exponent of 2.78, and this combination was then compared with the ratings of their respective composite pairs (*R*3). The figure shows four examples of combination sets and the average rating given to each of the image pairs by the 11 observers. Columns 1 and 2 show image pairs and ratings in which there was only one change in the image. Column 3 shows the composite image pair and associated rating (*R*3) where there were two image pairs. The final column (*R*3 estimated) shows the rating predicted for the composite pair by Minkowski summation of the ratings to the two component image pairs.

**Figure 4:**
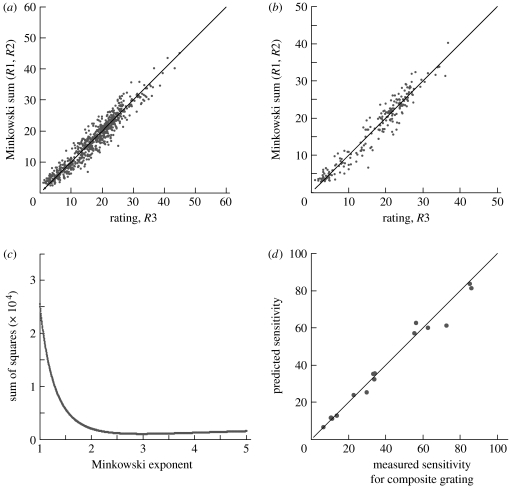
(*a*) For all 704 combination sets in the 3 experiments, the Minkowski sum (exponent=2.84) of the average ratings (*R*1 and *R*2) to the two component image pairs is plotted against the average measured rating (*R*3) for the respective composite image pair. The line of equality is shown. (*b*) The same as (*a*), but only for those 208 image pairs where the ratings (*R*1 and *R*2) to the component image pairs were within a factor of 1.4 of each other. The best Minkowski exponent is now 2.98. (*c*) The graph shows how the sum of squares deviation between the predicted and measured *R*3 depends on the Minkowski exponent used to make the prediction for the 208 selected combination sets. (*d*) Minkowski summation (exponent 1.97) of the contrast sensitivities for detecting 16 sinusoidal grating stimuli, which consisted of two spatially separate Gabor patches (see §4). Contrast sensitivity (reciprocal of Michelson contrast) was measured for small patches of grating using a two-interval forced-choice paradigm and staircase control of contrast. The observer viewed a central spot while Gabor patches (spread of 0.38°) could appear either 1.14° to the left of the spot or 1.14° to the right, or together. When presented together, the contrasts of the two patches were fixed in a ratio that prior experiment had suggested would make them approximately equally detectable. The graph shows the measured sensitivity to the paired presentation compared with a value predicted by calculation from the sensitivities to the two component stimuli presented singly. The left and right Gabor patches might have had the same or different spatial frequency and orientation. In some experiments, the patches were presented against a uniform grey background, but in others there was a masking pattern of static noise filtered to have a 1/*f* amplitude spectrum. Results for 16 combinations of left and right Gabor patches are shown.

**Table 1:** Summary statistics for the various models and datasets described in the text. (Pearson's correlation coefficients are shown. The rightmost column shows the sum of squared deviations between measured and predicted *R*3, divided by the number of points in the particular dataset.)

experiment	summation rule	number of sets	correlation coefficient	sum of squares
1	arithmetic sum	136	0.903	141.50
1	mean	136	0.903	54.03
1	maximum	136	0.902	15.67
1	Minkowski 2.78	136	0.929	7.60
2	arithmetic sum	136	0.832	74.15
2	mean	136	0.832	42.69
2	maximum	136	0.895	9.35
2	Minkowski 2.79	136	0.908	5.71
3	arithmetic sum	432	0.929	45.77
3	mean	432	0.929	33.30
3	maximum	432	0.954	5.96
3	Minkowski 2.95	432	0.963	4.19
1, 2, 3	maximum	704	0.947	8.49
1, 2, 3	Minkowski 2.84	704	0.960	5.14
subset	maximum	208	0.964	13.72
subset	Minkowski 2.98	208	0.967	4.94

## Supplementary Material

Additional materials and method Construction of stimuli; Observers; Reliability of rating measurements

## References

[bib1] Atick J.J, Redlich A.N (1992). What does the retina know about natural scenes?. Neural Comput.

[bib2] Bonneh Y, Sagi D (1998). Effects of spatial configuration on contrast detection. Vision Res.

[bib3] Bonneh Y, Sagi D (1999). Contrast integration across space. Vision Res.

[bib4] Bruce V, Langton S (1994). The use of pigmentation and shading in recognizing the sex and identities of faces. Perception.

[bib5] Campbell F.W, Robson J.G (1968). Application of Fourier analysis to the visibility of gratings. J. Physiol.

[bib6] Chirimuuta M, Clatworthy P.L, Tolhurst D.J (2003). Coding of the contrasts in natural images by visual cortex (V1) neurons: a Bayesian approach. J. Opt. Soc. Am. A.

[bib7] Clatworthy P.L, Chirimuuta M, Tolhurst D.J (2003). Coding of the contrasts in natural images by populations of neurons in striate visual cortex (V1). Vision Res.

[bib8] Ennis D.M, Palen J.J, Mullen K (1988). A multidimensional stochastic theory of similarity. J. Math. Psychol.

[bib9] Field D.J (1987). Relations between the statistics of natural images and the response properties of cortical cells. J. Opt. Soc. Am. A.

[bib10] Gescheider G.A (1997). Psychophysics—the fundamentals.

[bib11] Ghose G.M, Maunsell J (1999). Specialized representations in visual cortex: a role for binding?. Neuron.

[bib12] Graham N.V (1977). Visual detection of aperiodic spatial stimuli by probability summation among narrowband channels. Vision Res.

[bib13] Graham N.V (1989). Visual pattern analyzers.

[bib14] Graham N.V, Robson J.G, Nachmias J (1978). Grating summation in fovea and periphery. Vision Res.

[bib15] Haxby J.V, Ungerleider L.G, Clark V.P, Schouten J.L, Hoffman E.A, Martin A (1999). The effect of face inversion on activity in human neural systems for face and object perception. Neuron.

[bib16] King-Smith P.E, Kulikowski J.J (1975). Detection of gratings by independent activation of line detectors. J. Physiol.

[bib17] Koch C, Ullman S (1985). Shifts in selective visual attention: towards the underlying neural circuitry. Hum. Neurobiol.

[bib18] Koene A.R, Zhaoping L (2007). Feature-specific interactions in salience from combined feature contrasts: evidence for a bottom-up saliency map in V1. J. Vision.

[bib19] Lewis A, Zhaoping L (2005). Saliency from natural scene statistics. Program No. 821.11. 2005. Viewer/Itinerary Planner.

[bib20] Li Z (2002). A saliency map in primary visual cortex. Trends Cogn. Sci.

[bib21] Lovell P.G, Tolhurst D.J, Párraga C.A, Baddeley R, Leonards U, Troscianko J, Troscianko T (2005). On the stability of the color-opponent signals under changes of illuminant in natural scenes. J. Opt. Soc. Am. A.

[bib22] Lovell P.G, Párraga C.A, Ripamonti C, Troscianko T, Tolhurst D.J (2006). Evaluation of a multi-scale color model for visual difference prediction. ACM Trans. Appl. Percept.

[bib24] Meese T.S, Williams C.B (2000). Probability summation for multiple patches of luminance modulation. Vision Res.

[bib25] Meinhardt G, Persike M (2003). Strength of feature contrast mediates interaction among feature domains. Spat. Vision.

[bib27] Meinhardt G, Schmidt M, Persike M, Röers B (2004). Feature synergy depends on feature contrast and objecthood. Vision Res.

[bib26] Meinhardt G, Persike M, Mesenholl B, Hagemann C (2006). Cue combination in a combined feature contrast detection and figure identification task. Vision Res.

[bib28] Mostafavi H, Sakrison D.J (1976). Structure and properties of a single channel in human visual system. Vision Res.

[bib29] Párraga C.A, Troscianko T, Tolhurst D.J (2005). The effects of amplitude–spectrum statistics on foveal and peripheral discrimination of changes in natural images, and a multiresolution model. Vision Res.

[bib30] Pelli D.G, Zhang L (1991). Accurate control of contrast on microcomputer displays. Vision Res.

[bib31] Quick R.F (1974). A vector magnitude model of contrast detection. Kybernetik.

[bib32] Quick R.F, Mullins W.W, Reichert T.A (1978). Spatial summation effects on 2-component grating thresholds. J. Opt. Soc. Am.

[bib33] Robson J.G, Graham N.V (1981). Probability summation and regional variation in contrast sensitivity across the visual field. Vision Res.

[bib34] Rohaly A.M, Ahumada A.J, Watson A.B (1997). Object detection in natural backgrounds predicted by discrimination performance and models. Vision Res.

[bib35] Rossion B, Gauthier I, Goffaux V, Tarr M.J, Crommelinck M (2002). Expertise training with novel objecys leads to left-lateralized facelike electrophysiological responses. Psychol. Sci.

[bib36] Schwartz O, Simoncelli E.P (2001). Natural signal statistics and sensory gain control. Nat. Neurosci.

[bib37] Shepard R.N (1964). Attention and the metric structure of stimulus space. J. Math. Psychol.

[bib38] Shepard R.N (1987). Toward a universal law of generalization for psychological science. Science.

[bib39] Schneidman E, Berry M.J, Segev R, Bialek W (2006). Weak pairwise correlations imply strongly correlated network states in a neural population. Nature.

[bib40] Simons D.J, Rensink R.A (2005). Change blindness: past, present, and future. Trends Cogn. Sci.

[bib41] Srinivasan M.V, Laughlin S.B, Dubs A (1982). Predictive coding: a fresh view of inhibition in the retina. Proc. R. Soc. B.

[bib42] Stromeyer C.F, Klein S (1975). Evidence against narrow-band spatial frequency channels in human vision: detectability of frequency modulated gratings. Vision Res.

[bib43] Stevens S.S (1975). Psychophysics: introduction to its perceptual, neural, and social prospects.

[bib44] Titchener E.B (1908). Lectures on the elementary psychology of feeling and attention.

[bib45] To M, Lovell P.G, Troscianko T, Tolhurst D.J (2006). Summation of suprathreshold cues in complex visual discriminations using natural scene stimuli. Perception.

[bib46] To M, Lovell P.G, Troscianko T, Tolhurst D.J (2007). Minkowski summation of cues in complex visual discriminations using natural scene stimuli. J. Vis.

[bib47a] To, M., Troscianko, T. & Tolhurst, D. J. In preparation. A general rule for perceptual feature integration in visual scenes, musical sequences and phonetic utterances.

[bib47] Tolhurst D.J (1975). Reaction times in the detection of gratings by human observers: a probabilistic mechanism. Vision Res.

[bib48] Tolhurst, D. J., Párraga, C. A., Lovell, P. G., Ripamonti, C. & Troscianko, T. 2005 A multiresolution color model for visual difference prediction. In *Proc. 2nd Conference of APGV* ACM International Conference Proceeding Series 95, pp. 135–138.

[bib49] Treisman A (1996). The binding problem. Curr. Opin. Neurobiol.

[bib50] Vinje W.E, Gallant J.L (2000). Sparse coding and decorrelation in primary visual cortex during natural vision. Science.

[bib23] von der Malsburg C (1995). Binding in models of perception and brain function. Curr. Opin. Neurobiol.

[bib51] Vuong Q.C, Peissig J.J, Harrison M.C, Tarr M.J (2005). The role of surface pigmentation for recognition revealed by contrast reversal in faces and Greebles. Vision Res.

[bib52] Watson A.B (1982). Summation of grating patches indicates many types of detector at one retinal location. Vision Res.

[bib53] Watson A.B, Ahumada A.J (2005). A standard model for foveal detection of spatial contrast. J. Vis.

[bib54] Watson A.B, Nachmias J (1980). Summation of asynchronous gratings. Vision Res.

[bib55] Watson A.B, Solomon J.A (1997). Model of visual contrast gain control and pattern masking. J. Opt. Soc. Am. A.

[bib56] Wilson H.R, Gelb D.J (1984). Modified line-element theory for spatial-frequency and width discrimination. J. Opt. Soc. Am. A.

[bib57] Wolfe J.M, Cave K.R (1999). The psychophysical evidence for a binding problem in human vision. Neuron.

[bib58] Yin R.K (1969). Looking at upside-down faces. J. Exp. Psychol.

[bib59] Zhaoping L, May K.A (2007). Psychophysical tests of the hypothesis of a bottom-up saliency map in the primary visual cortex. PLoS Comput. Biol.

