# Supplementary material for: Summation of perceptual cues in natural visual scenes
Source: Proc Biol Sci. 2008 Jul 15;275(1649):2299–308. doi: 10.1098/rspb.2008.0692 (PMC2495046; doi:10.1098/rspb.2008.0692)
Supplement: Supplementary file 1 [file rspb20080692s01.doc]

# Construction of Stimuli

The images from the Nikon still cameras were taken with a fixed, small aperture and with the white balance setting at ‘cloudy’; they were retrieved as uncompressed tiff files. The spectral sensitivity of R, G and B sensors of both Nikon cameras was fully characterized, including estimates of the nonlinear relation between pixel value and luminance input for the R, G and B sensors on the cloudy setting. The camcorder video stream was saved on tape (where it was presumably compressed); single frames were then played back through a firewire interface and they were digitized on a PC as 720×576 pixels. This resolution was for a pair of interleaved frames and, when there was considerable movement in the scenes, only alternate lines could be used in any one frame. For the JVC camcorder, we measured only the relationships between pixel value and luminance for the R, G and B planes. The images from all cameras were manipulated on computer with the nonlinear gamma given to us by the cameras; but before display on the linearized CRT, the nonlinearities between pixel value and luminance for each camera were corrected for. We did not compensate for any differences in the spectral sensitivities of the cameras’ sensors and the CRT’s phosphors. To get from full-sized images to the 256×256 pixel stimuli, the originals were cropped, or were subsampled by taking every nth column and row, and then cropped.

# Observers

Experiments 1 and 2 were performed on two different groups of 11 observers, all of whom were ignorant of the rationale for the experiments. There were in total 14 female and 8 male observers: 7 females and 4 males in each condition. Fifteen observers (8 female, 7 male) were tested in Experiment 3: while some had previously participated in other rating experiments, they remained naive to the purpose of this experiment. To ensure that all observers had normal vision (with their usual corrections where appropriate), they were screened using a Snellen Letter chart and the Ishihara colour test (10th edn).

# Reliability of rating measurements

The present experiments rely on magnitude estimation ratings. Although these may seem to be subjective judgements, they did provide reliable measurements. We have taken several steps to verify this. Ideally, we would want to examine within-observer and between-observer consistency.

(i) *Within-observer consistency*

In pilot studies (not presented in this paper), we asked observers to complete a rating task twice. In the first, two observers were presented with 450 image pairs twice (a subset of the 900 image pairs presented in experiment 1). The correlation coefficients Pearson’s *r*) between their first and second runs were 0.79 in both cases. In a second pilot study, seven naive observers rated 180 upright image pairs and their 180 inverted counterparts presented in random order (also subsets from experiment 1). When five observers completed the task a second time, the correlation between their ratings from run 1 and run 2 ranged between 0.64 and 0.80, with an average of 0.72 (a value similar to that found in the first pilot). Incidentally, the correlation between each observer’s ratings for upright and inverted stimuli was 0.74 on average. Finally, three observers repeated experiment 2 (all 900 stimuli), at an interval of three months; the correlation between their ratings on run 1 and 2 averaged 0.69.

We were concerned that, if we asked observers to repeat experiments, they might begin to recognize image pairs and then remember their previous ratings, rather than make new ratings. However, with that caveat, our findings do suggest that, when observers perform the same or similar experiments twice, the correlation between their ratings in the two cases will be approximately −0.74; this is pleasingly high, given that the observers were presented with such a variety of image changes along many disparate dimensions, and our observers were given the freedom to choose any positive integer for their subjective ratings.

(ii) *Between-observer consistency*

In all our experiments, we were able to compare the ratings of each observer for a given stimulus set with the ratings given by each other observer to the same set. For the experiments reported here, the between-observer correlation coefficients were on average, as follows: 0.59 experiment 1), 0.55 (experiment 2) and 0.67 experiment 3). That these values are lower than the within-observer correlations implies that different observers may maintain different (though consistent) rating scales (e.g. Zwislocki 1983; Gescheider 1997).

(iii) *Using across-observer averages*

For each experiment, we have collected ratings from 11 or 15 observers (one run each) and have averaged together their ratings for subsequent analysis. Averaging together the ratings of 10 or more observers produces datasets that are robust. For instance, in experiment 3, the average of the 15 observers’ ratings for upright images had a correlation coefficient of 0.97 with the average of their ratings for the inverted counterparts. In the pilot experiment where seven observers viewed 180 upright and inverted versions of the same image pairs taken from experiment 1, the correlation between averaged upright and averaged inverted rating was 0.88. Lastly, we performed a variant of experiment 1 with 10 new observers (the stimuli were presented for only 100 ms instead of 833 ms); the average of 11 observers’ ratings for the 833 ms stimuli had a correlation coefficient of 0.90 with the average of the 10 observers’ ratings for the 100 ms stimuli.

Reference

Zwislocki, J. J. 1983 Group and individual relations between sensation magnitudes and their numerical estimates. *Percept. Psychophys.* **33**, 460−468.
